# The *δ*(2,2)-Invariant on Statistical Submanifolds in Hessian Manifolds of Constant Hessian Curvature

**DOI:** 10.3390/e22020164

**Published:** 2020-01-31

**Authors:** Adela Mihai, Ion Mihai

**Affiliations:** 1 Department of Mathematics and Computer Science, Technical University of Civil Engineering Bucharest, 020396 Bucharest, Romania; adela.mihai@utcb.ro; 2 Department of Mathematics, Faculty of Mathematics and Computer Science, University of Bucharest, 010014 Bucharest, Romania

**Keywords:** statistical manifolds, Hessian manifolds, Hessian sectional curvature, scalar curvature, Ricci curvature, Chen inequality

## Abstract

We establish Chen inequality for the invariant δ(2,2) on statistical submanifolds in Hessian manifolds of constant Hessian curvature. Recently, in co-operation with Chen, we proved a Chen first inequality for such submanifolds. The present authors previously initiated the investigation of statistical submanifolds in Hessian manifolds of constant Hessian curvature; this paper represents a development in this topic.

## 1. Introduction

In 1985, Amari [1] introduced the notion of a statistical manifold, closely related with information geometry. At the same time, the geometry of statistical manifolds is not far from affine differential geometry, because it also involves dual connections (also called conjugate connections). A Hessian structure is a particular case of a statistical structure.

Opozda, in [2], defined a sectional curvature on a statistical manifold, which cannot be defined in a standard way (as in Riemannian geometry), because the dual connections are not metric.

The main Riemannian invariants are the curvature invariants, with many important applications, for example in physics. Among them, the most studied and well known are the sectional curvature, scalar curvature, and Ricci curvatures.

In submanifold theory, beside the study of the geometric properties of submanifolds, establishing sharp relationships between intrinsic and extrinsic invariants is another topic of interest.

In 1993 [3], Chen defined a new type of curvature invariants, which he called δ-invariants (or Chen invariants) (see also [4,5]). In the same paper, he proved the Chen first inequality for submanifolds in Riemannian space forms. The Chen first invariant of an *n*-dimensional Riemannian manifold $M^{n}$ is defined by $\delta_{M^{n}}=\tau -\inf K$, where τ and *K* are the scalar and sectional curvatures of $M^{n}$, respectively.

Moreover, $\delta \left(2,2\right)\left(p\right)=\tau \left(p\right)-\inf[K\left(\pi_{1}\right)+K\left(\pi_{2}\right)]$, where $\pi_{1}$ and $\pi_{2}$ are mutually orthogonal plane sections at $p\in M^{n}$. Chen and his coworkers studied this invariant for Lagrangian submanifolds in complex space forms (see [6,7]).

On the other hand, in [8], it is shown that a Hessian manifold of constant Hessian curvature *c* is a statistical manifold of null constant curvature and a Riemannian space form of constant sectional curvature −c/4 (with respect to the sectional curvature defined by the Levi-Civita connection). The present authors (see [9]) initiated the study of statistical submanifolds in such manifolds. In the same paper [9], we established a Euler inequality and also a Chen–Ricci inequality.

The curvature invariants of statistical submanifolds in different ambient spaces were recently studied by several authors, for example in Kenmotsu statistical manifolds of constant ϕ-sectional curvature (see [10]). Also, a generalized Wintgen inequality for statistical submanifolds was obtained in [11].

In 2019, Chen and the present authors [12] proved a Chen first inequality for statistical submanifolds in Hessian manifolds of constant Hessian curvature.

The main goal of this paper is to establish a Chen-like inequality for the invariant δ(2,2) on such submanifolds.

## 2. Statistical Manifolds and Statistical Submanifolds

A *statistical manifold* is an *m*-dimensional Riemannian manifold $\left({\tilde{M}}^{m},g\right)$ endowed with a pairing of torsion-free affine connections $\tilde{\nabla }$ and ${\tilde{\nabla }}^{*}$ satisfying

(1) $$\begin{array}{c} Z\tilde{g}\left(X,Y\right)=\tilde{g}\left({\tilde{\nabla }}_{Z}X,Y\right)+\tilde{g}\left(X,{\tilde{\nabla }}_{Z}^{*}Y\right), \end{array}$$

for any $X,Y,Z\in \Gamma (T{\tilde{M}}^{m}).$ One says that the connections $\tilde{\nabla }$ and ${\tilde{\nabla }}^{*}$ are *dual connections* (see [1,13,14]); one has ${\left({\tilde{\nabla }}^{*}\right)}^{*}=\tilde{\nabla }.$ The pairing $\left(\tilde{\nabla },g\right)$ is said to be a *statistical structure*.

The dual connection of a torsion-free affine connection $\tilde{\nabla }$ always exists and is given by

(2) $$\begin{array}{c} \tilde{\nabla }+{\tilde{\nabla }}^{*}=2{\tilde{\nabla }}^{0}, \end{array}$$

where ${\tilde{\nabla }}^{0}$ is the Levi-Civita connection on ${\tilde{M}}^{m}$.

The curvature tensor fields with respect to the dual connections $\tilde{\nabla }$ and ${\tilde{\nabla }}^{*}$ are denoted by $\tilde{R}$ and ${\tilde{R}}^{*},$ respectively.

The curvature tensor ${\tilde{R}}^{0}$ associated with ${\tilde{\nabla }}^{0}$ is known as the Riemannian curvature tensor.

A statistical structure $\left(\tilde{\nabla },g\right)$ is called *of constant curvature* ε∈R [14] if

(3) $$\begin{array}{c} \tilde{R}\left(X,Y\right)Z=\varepsilon \left\{g\left(Y,Z\right)X-g\left(X,Z\right)Y\right\},\;\forall X,Y,Z\in \Gamma \left(T{\tilde{M}}^{m}\right). \end{array}$$

If the constant curvature is 0, then it is known as a *Hessian structure.*

The curvature tensor fields $\tilde{R}$ and ${\tilde{R}}^{*}$ of the dual connections are related by

(4) $$\begin{array}{c} g\left({\tilde{R}}^{*}\left(X,Y\right)Z,W\right)=-g\left(Z,\tilde{R}\left(X,Y\right)W\right). \end{array}$$

It is clear that if $\left(\tilde{\nabla },g\right)$ is a statistical structure of constant curvature ε, then $\left({\tilde{\nabla }}^{*},g\right)$ is also a statistical structure of constant curvature ε (obviously, if $\left(\tilde{\nabla },g\right)$ is Hessian, $\left({\tilde{\nabla }}^{*},g\right)$ is also Hessian [8]).

On a Hessian manifold (${\tilde{M}}^{m},\tilde{\nabla })$, denote by $\gamma =\tilde{\nabla }-{\tilde{\nabla }}^{0}$. The tensor field $\tilde{Q}$ of type (1,3) defined by

$$\tilde{Q}\left(X,Y\right)=\left[\gamma_{X},\gamma_{Y}\right],\;X,Y\in \Gamma \left(T{\tilde{M}}^{m}\right)$$

is said to be the *Hessian curvature tensor* for $\tilde{\nabla }$ (see [2,8]). One has

$$\tilde{R}\left(X,Y\right)+{\tilde{R}}^{*}\left(X,Y\right)=2{\tilde{R}}^{0}\left(X,Y\right)+2\tilde{Q}\left(X,Y\right).$$

Then, the Hessian sectional curvatures can be defined on a Hessian manifold using $\tilde{Q}$. More precisely, if one considers $p\in {\tilde{M}}^{m}$ and π a plane section in $T_{p}{\tilde{M}}^{m}$ and an orthonormal basis {X,Y} of π, then the Hessian sectional curvature is defined by

$$\tilde{K}\left(\pi \right)=g\left(\tilde{Q}\left(X,Y\right)Y,X\right),$$

independent of the choice of an orthonormal basis.

A Hessian manifold is of constant Hessian sectional curvature *c* if and only if (see [8])

$$\tilde{Q}\left(X,Y,Z,W\right)=\frac{c}{2}\left\{g\left(X,Y\right)g\left(Z,W\right)+g\left(X,W\right)g\left(Y,Z\right)\right\},$$

for all vector fields on ${\tilde{M}}^{m}$.

In [8], it is proved that a Hessian manifold of constant Hessian sectional curvature *c* is a Riemannian space form of constant sectional curvature −c/4.

Let $\left({\tilde{M}}^{m},g\right)$ be a statistical manifold and $M^{n}$ a submanifold of ${\tilde{M}}^{m}$ of dimension *n*. The induced connections *∇* and $\nabla^{*}$ and the induced metric *g* define a statistical structure on the submanifold $\left(M^{n},g\right)$. The set of the sections of the normal bundle to $M^{n}$ is denoted by $\Gamma (T^{⊥}M^{n})$.

The corresponding Gauss formulae for the conjugate connections (see [15]) are

(5) $$\begin{array}{c} {\tilde{\nabla }}_{X}Y=\nabla_{X}Y+h\left(X,Y\right), \end{array}$$

(6) $$\begin{array}{c} {\tilde{\nabla }}_{X}^{*}Y=\nabla_{X}^{*}Y+h^{*}\left(X,Y\right), \end{array}$$

for any $X,Y\in \Gamma (TM^{n})$; *h*, $h^{*}:\Gamma \left(TM^{n}\right)\times \Gamma \left(TM^{n}\right)\to \Gamma \left(T^{⊥}M^{n}\right)$ are symmetric and bilinear and they are known as the *imbedding curvature tensor* of $M^{n}$ in ${\tilde{M}}^{m}$ for $\tilde{\nabla }$ and the *imbedding curvature tensor* of $M^{n}$ in ${\tilde{M}}^{m}$ for ${\tilde{\nabla }}^{*},$ respectively. One remarks that (∇,g) and $\left(\nabla^{*},g\right)$ are dual statistical structures on $M^{n}.$

Since *h* and $h^{*}$ are bilinear, there exist linear transformations $A_{\xi }$ and $A_{\xi }^{*}$ on $TM^{n}$ defined by

(7) $$\begin{array}{c} g\left(A_{\xi }X,Y\right)=g\left(h\left(X,Y\right),\xi \right), \end{array}$$

(8) $$\begin{array}{c} g\left(A_{\xi }^{*}X,Y\right)=g\left(h^{*}\left(X,Y\right),\xi \right), \end{array}$$

for any $\xi \in \Gamma (T^{⊥}M^{n})$ and $X,Y\in \Gamma (TM^{n}).$

Furthermore, the Weingarten formulae are [15]

(9) $$\begin{array}{c} {\tilde{\nabla }}_{X}\xi =-A_{\xi }^{*}X+\nabla_{X}^{⊥}\xi , \end{array}$$

(10) $$\begin{array}{c} {\tilde{\nabla }}_{X}^{*}\xi =-A_{\xi }X+\nabla_{X}^{*⊥}\xi , \end{array}$$

for any $\xi \in \Gamma (T^{⊥}M^{n})$ and $X\in \Gamma (TM^{n}).$ The connections $\nabla^{⊥}$ and $\nabla^{*⊥}$ defined by Equations (9) and (10) are Riemannian dual connections with respect to the induced metric on $\Gamma (T^{⊥}M^{n}).$

Let $\left\{e_{1},\ldots ,e_{n}\right\}$ and $\left\{e_{n+1},\ldots ,e_{m}\right\}$ be orthonormal tangent and normal frames on *M*, respectively. Then, the mean curvature vector fields are defined by

$$H=\frac{1}{n}\sum_{i=1}^{n}h\left(e_{i},e_{i}\right)=\frac{1}{n}\sum_{\alpha =n+1}^{m}\left(\sum_{i=1}^{n}h_{ii}^{\alpha }\right)e_{\alpha },\;h_{ij}^{\alpha }=g\left(h\left(e_{i},e_{j}\right),e_{\alpha }\right),$$

$$H^{*}=\frac{1}{n}\sum_{i=1}^{n}h^{*}\left(e_{i},e_{i}\right)=\frac{1}{n}\sum_{\alpha =n+1}^{m}\left(\sum_{i=1}^{n}h_{ii}^{*\alpha }\right)e_{\alpha },h_{ij}^{*\alpha }=g\left(h^{*}\left(e_{i},e_{j}\right),e_{\alpha }\right),$$

for 1≤i,j≤n and n+1≤α≤m.

The Gauss, Codazzi, and Ricci equations for statistical submanifolds, with respect to the dual connections, were established by Vos [15].

**Proposition** **1.** *[15] Let $\tilde{\nabla }$ and ${\tilde{\nabla }}^{*}$ be dual connections on a statistical manifold ${\tilde{M}}^{m}$, and let *∇* be the induced connection by $\tilde{\nabla }$ on a statistical submanifold $M^{n}.$ Let $\tilde{R}$ and R be the Riemannian curvature tensors for $\tilde{\nabla }$ and ∇, respectively. Then,*

(11) $$\begin{array}{cc} g\left(\tilde{R}\left(X,Y\right)Z,W\right)= & g\left(R\left(X,Y\right)Z,W\right)+g\left(h\left(X,Z\right),h^{*}\left(Y,W\right)\right)\\ & -g(h^{*}\left(X,W\right),h\left(Y,Z\right)), \end{array}$$

(12) $$\begin{array}{cc} {\left(\tilde{R}\left(X,Y\right)Z\right)}^{⊥}= & \nabla_{X}^{⊥}h\left(Y,Z\right)-h\left(\nabla_{X}Y,Z\right)-h\left(Y,\nabla_{X}Z\right)\\ & -\{\nabla_{Y}^{⊥}h\left(Y,Z\right)-h\left(\nabla_{Y}X,Z\right)-h\left(X,\nabla_{Y}Z\right)\}, \end{array}$$

(13) $$\begin{array}{c} g\left(R^{⊥}\left(X,Y\right)\xi ,\eta \right)=g\left(\tilde{R}\left(X,Y\right)\xi ,\eta \right)+g\left(\left[A_{\xi }^{*},A_{\eta }\right]X,Y\right), \end{array}$$

*where $R^{⊥}$ is the Riemannian curvature tensor of $\nabla^{⊥}$ on $T^{⊥}M^{n},$$\xi ,\eta \in \Gamma (T^{⊥}M^{n})$ and $\left[A_{\xi }^{*},A_{\eta }\right]=A_{\xi }^{*}A_{\eta }-A_{\eta }A_{\xi }^{*}.$*

**Proposition** **2.** *[15] Let $\tilde{\nabla }$ and ${\tilde{\nabla }}^{*}$ be dual connections on a statistical manifold ${\tilde{M}}^{m}$, and let $\nabla^{*}$ be the induced connection by ${\tilde{\nabla }}^{*}$ on a statistical submanifold $M^{n}.$ Let ${\tilde{R}}^{*}$ and $R^{*}$ be the Riemannian curvature tensors for ${\tilde{\nabla }}^{*}$ and $\nabla^{*},$ respectively. Then,*

(14) $$\begin{array}{cc} g\left({\tilde{R}}^{*}\left(X,Y\right)Z,W\right)= & g\left(R^{*}\left(X,Y\right)Z,W\right)+g\left(h^{*}\left(X,Z\right),h\left(Y,W\right)\right)\\ & -g(h\left(X,W\right),h^{*}\left(Y,Z\right)), \end{array}$$

(15) $$\begin{array}{cc} {\left({\tilde{R}}^{*}\left(X,Y\right)Z\right)}^{⊥}= & \nabla_{X}^{*⊥}h^{*}\left(Y,Z\right)-h^{*}\left(\nabla_{X}^{*}Y,Z\right)-h^{*}\left(Y,\nabla_{X}^{*}Z\right)\\ & -\{\nabla_{Y}^{*⊥}h^{*}\left(Y,Z\right)-h^{*}\left(\nabla_{Y}^{*}X,Z\right)-h^{*}\left(X,\nabla_{Y}^{*}Z\right)\}, \end{array}$$

(16) $$\begin{array}{c} g\left(R^{*⊥}\left(X,Y\right)\xi ,\eta \right)=g\left({\tilde{R}}^{*}\left(X,Y\right)\xi ,\eta \right)+g\left(\left[A_{\xi },A_{\eta }^{*}\right]X,Y\right), \end{array}$$

*where $R^{*⊥}$ is the Riemannian curvature tensor of $\nabla^{⊥*}$ on $T^{⊥}M^{n},$ξ,η∈$\Gamma (T^{⊥}M^{n})$ and $\left[A_{\xi },A_{\eta }^{*}\right]=A_{\xi }A_{\eta }^{*}-A_{\eta }^{*}A_{\xi }.$*

## 3. Chen Inequality for the Chen Invariant δ(2,2)

In [9], the present authors proved a Euler inequality and also a Chen–Ricci inequality for submanifolds in a Hessian manifold of constant Hessian curvature. Recently Chen and the present authors [12] obtained a Chen first inequality for such submanifolds. Herein, we establish a Chen inequality for the Chen invariant δ(2,2) on statistical submanifolds in Hessian manifolds of constant Hessian curvature. We state the following algebraic lemma which is used in the proof of our main result.

**Lemma** **1.** *Let n≥4 be an integer and let $a_{1},\ldots ,a_{n}$ be n real numbers. Then, one has*

$$\sum_{1\leq i<j\leq n}a_{i}a_{j}-a_{1}a_{2}-a_{3}a_{4}\leq \frac{n-3}{2(n-2)}{\left(\sum_{i=1}^{n}a_{i}\right)}^{2}.$$

*Moreover, the equality holds if and only if $a_{1}+a_{2}=a_{3}+a_{4}=a_{5}=\ldots =a_{n}$.*

**Proof.**  We shall prove this Lemma by mathematical induction.

For n=4, the inequality becomes

$$a_{1}a_{3}+a_{1}a_{4}+a_{2}a_{3}+a_{2}a_{4}\leq \frac{1}{4}{\left(a_{1}+a_{2}+a_{3}+a_{4}\right)}^{2},$$

or equivalently $0\leq {\left(a_{1}+a_{2}-a_{3}-a_{4}\right)}^{2}$, with the equality holding if and only if $a_{1}+a_{2}=a_{3}+a_{4}$.

Let us put

(17) $$\begin{array}{c} L_{n}=\sum_{1\leq i<j\leq n}a_{i}a_{j}-a_{1}a_{2}-a_{3}a_{4} \end{array}$$

and assume

(18) $$\begin{array}{c} P\left(n\right):\;L_{n}\leq \frac{n-3}{2(n-2)}{\left(\sum_{i=1}^{n}a_{i}\right)}^{2}. \end{array}$$

The equality sign of Equation (18) holds if and only if $a_{1}+a_{2}=a_{3}+a_{4}=a_{5}=\ldots =a_{n}$.

By using P(n), we find

(19) $$\begin{array}{cc} L_{n+1} & =L_{n}+\left(a_{1}+\ldots +a_{n}\right)a_{n+1}\\ & \leq \frac{n-3}{2(n-2)}{\left(a_{1}+\ldots +a_{n}\right)}^{2}+\left(a_{1}+\ldots +a_{n}\right)a_{n+1}. \end{array}$$

On the other hand, obviously one has

(20) $$\begin{array}{cc} \frac{n-3}{2(n-2)}{\left(a_{1}+\ldots +a_{n}\right)}^{2} & +\left(a_{1}+a_{2}+\ldots +a_{n}\right)a_{n+1}\\ & \leq \frac{n-2}{2(n-1)}{\left(a_{1}+\ldots +a_{n}+a_{n+1}\right)}^{2}, \end{array}$$

because it it equivalent to

$${\left[\left(n-2\right)a_{n+1}-\left(a_{1}+\ldots +a_{n}\right)\right]}^{2}\geq 0.$$

Inequalities (18) and (20) imply P(n+1), with the equality sign holding if and only if we have equalities in Equations (19) and (20), i.e.,

$$\begin{array}{c} a_{1}+a_{2}=a_{3}+a_{4}=a_{5}=\ldots =a_{n},\\ \left(n-2\right)a_{n+1}=a_{1}+\ldots +a_{n}. \end{array}$$

Thus, $a_{n+1}=a_{n}$ and the proof is complete. ☐

Let ${\tilde{M}}^{m}\left(c\right)$ be a Hessian manifold of constant Hessian curvature *c*. Then, it is flat with respect to the dual connections $\tilde{\nabla }$ and ${\tilde{\nabla }}^{*}$. Moreover, ${\tilde{M}}^{m}\left(c\right)$ is a Riemannian space form of constant sectional curvature −c/4 (with respect to the Levi-Civita connection ${\tilde{\nabla }}^{0}$).

Let $M^{n}$ be an *n*-dimensional statistical submanifold of ${\tilde{M}}^{m}\left(c\right)$ and $p\in M^{n}$, and $\pi_{1}$ and $\pi_{2}$ mutually orthogonal plane sections at *p*. Consider orthonormal bases $\left\{e_{1},e_{2}\right\}$ of $\pi_{1}$, $\left\{e_{3},e_{4}\right\}$ of $\pi_{2}$, and $\left\{e_{1},\ldots ,e_{n}\right\}$ and $\left\{e_{n+1},\ldots ,e_{m}\right\}$ orthonormal bases of $T_{p}M^{n}$ and $T_{p}^{⊥}M^{n}$, respectively. We denote by $K_{0}$ the sectional curvature of the Levi-Civita connection $\nabla^{0}$ on $M^{n}$ and by $h^{0}$ the second fundamental form of $M^{n}$.

The sectional curvatures $K(\pi_{1})$ and $K(\pi_{2})$ of the plane sections $\pi_{1}$ and $\pi_{2}$, respectively, are

$$K\left(\pi_{1}\right)=\frac{1}{2}\left[g\left(R\left(e_{1},e_{2}\right)e_{2},e_{1}\right)+g\left(R^{*}\left(e_{1},e_{2}\right)e_{2},e_{1}\right)-2g\left(R^{0}\left(e_{1},e_{2}\right)e_{2},e_{1}\right)\right],$$

$$K\left(\pi_{2}\right)=\frac{1}{2}\left[g\left(R\left(e_{3},e_{4}\right)e_{4},e_{3}\right)+g\left(R^{*}\left(e_{3},e_{4}\right)e_{4},e_{3}\right)-2g\left(R^{0}\left(e_{3},e_{4}\right)e_{4},e_{3}\right)\right].$$

Using Equations (11) and (14), we get

$$\begin{array}{cc} K(\pi_{1}) & =\frac{1}{2}[g\left(h^{*}\left(e_{1},e_{1}\right),h\left(e_{2},e_{2}\right)\right)+g\left(h\left(e_{1},e_{1}\right),h^{*}\left(e_{2},e_{2}\right)\right)\\ & \;-2g\left(h\left(e_{1},e_{2}\right),h^{*}\left(e_{1},e_{2}\right)\right)]-K_{0}\left(\pi_{1}\right)\\ & =\frac{1}{2}\sum_{\alpha =n+1}^{m}\left(h_{11}^{*\alpha }h_{22}^{\alpha }+h_{11}^{\alpha }h_{22}^{*\alpha }-2h_{12}^{\alpha }h_{12}^{*\alpha }\right)-K_{0}\left(\pi_{1}\right)\\ & =\frac{1}{2}\sum_{\alpha =n+1}^{m}[\left(h_{11}^{\alpha }+h_{11}^{*\alpha }\right)\left(h_{22}^{\alpha }+h_{22}^{*\alpha }\right)-h_{11}^{\alpha }h_{22}^{\alpha }-h_{11}^{*\alpha }h_{22}^{*\alpha }\\ & \;-{\left(h_{12}^{\alpha }+h_{12}^{*\alpha }\right)}^{2}+{\left(h_{12}^{\alpha }\right)}^{2}+{\left(h_{12}^{*\alpha }\right)}^{2}]-K_{0}\left(\pi_{1}\right)\\ & =\sum_{\alpha =n+1}^{m}\{2\left[h_{11}^{0\alpha }h_{22}^{0\alpha }-{\left(h_{12}^{0\alpha }\right)}^{2}\right]-\frac{1}{2}\left[h_{11}^{\alpha }h_{22}^{\alpha }-{\left(h_{12}^{\alpha }\right)}^{2}\right]\\ & \;-\frac{1}{2}\left[h_{11}^{*\alpha }h_{22}^{*\alpha }-{\left(h_{12}^{*\alpha }\right)}^{2}\right]\}-K_{0}\left(\pi_{1}\right). \end{array}$$

The equation of Gauss for the Levi-Civita connection implies

(21) $$\begin{array}{cc} K(\pi_{1})= & \frac{c}{2}+K_{0}\left(\pi_{1}\right)-\frac{1}{2}\sum_{\alpha =n+1}^{m}\left[h_{11}^{\alpha }h_{22}^{\alpha }-{\left(h_{12}^{\alpha }\right)}^{2}\right]\\ & -\frac{1}{2}\sum_{\alpha =n+1}^{m}\left[h_{11}^{*\alpha }h_{22}^{*\alpha }-{\left(h_{12}^{*\alpha }\right)}^{2}\right]. \end{array}$$

Analogously, we have

(22) $$\begin{array}{cc} K(\pi_{2})= & \frac{c}{2}+K_{0}\left(\pi_{2}\right)-\frac{1}{2}\sum_{\alpha =n+1}^{m}\left[h_{33}^{\alpha }h_{44}^{\alpha }-{\left(h_{34}^{\alpha }\right)}^{2}\right]\\ & -\frac{1}{2}\sum_{\alpha =n+1}^{m}\left[h_{33}^{*\alpha }h_{44}^{*\alpha }-{\left(h_{34}^{*\alpha }\right)}^{2}\right]. \end{array}$$

On the other hand, let τ be the scalar curvature of $M^{n}$ (with respect to the Hessian curvature tensor *Q*). Then, from Equations (11) and (14), we have

$$\begin{array}{cc} \tau & =\frac{1}{2}\sum_{1\leq i<j\leq n}\left[g\left(R\left(e_{i},e_{j}\right)e_{j},e_{i}\right)+g\left(R^{*}\left(e_{i},e_{j}\right)e_{j},e_{i}\right)-2g\left(R^{0}\left(e_{i},e_{j}\right)e_{j},e_{i}\right)\right]\\ & =\frac{1}{2}\sum_{1\leq i<j\leq n}[g\left(h^{*}\left(e_{i},e_{i}\right),h\left(e_{j},e_{j}\right)\right)+g\left(h\left(e_{i},e_{i}\right),h^{*}\left(e_{j},e_{j}\right)\right)\\ & \;-2g\left(h\left(e_{i},e_{j}\right),h^{*}\left(e_{i},e_{j}\right)\right)]-\tau_{0}\\ & =\frac{1}{2}\sum_{\alpha =n+1}^{m}\sum_{1\leq i<j<n}\left(h_{ii}^{*\alpha }h_{jj}^{\alpha }+h_{ii}^{\alpha }h_{jj}^{*\alpha }-2h_{ij}^{\alpha }h_{ij}^{*\alpha }\right)-\tau_{0}\\ & =\frac{1}{2}\sum_{\alpha =n+1}^{m}\sum_{1\leq i<j\leq n}[\left(h_{ii}^{\alpha }+h_{ii}^{*\alpha }\right)\left(h_{jj}^{\alpha }+h_{jj}^{*\alpha }\right)-h_{ii}^{\alpha }h_{jj}^{\alpha }-h_{ii}^{*\alpha }h_{jj}^{*\alpha }\\ & \;-{\left(h_{ij}^{\alpha }+h_{ij}^{*\alpha }\right)}^{2}+{\left(h_{ij}^{\alpha }\right)}^{2}+{\left(h_{ij}^{*\alpha }\right)}^{2}]-\tau_{0}\\ & =\sum_{\alpha =n+1}^{m}\sum_{1\leq i<j\leq n}\{2\left[h_{ii}^{0\alpha }h_{jj}^{0\alpha }-{\left(h_{ij}^{0\alpha }\right)}^{2}\right]-\frac{1}{2}\left[h_{ii}^{\alpha }h_{jj}^{\alpha }-{\left(h_{ij}^{\alpha }\right)}^{2}\right]\\ & \;-\frac{1}{2}\left[h_{ii}^{*\alpha }h_{jj}^{*\alpha }-{\left(h_{ij}^{*\alpha }\right)}^{2}\right]\}-\tau_{0}. \end{array}$$

In addition, the equation of Gauss for the Levi-Civita connection implies

(23) $$\begin{array}{cc} \tau = & \;\tau_{0}+n\left(n-1\right)\frac{c}{4}-\frac{1}{2}\sum_{\alpha =n+1}^{m}\sum_{1\leq i<j\leq n}\left[h_{ii}^{\alpha }h_{jj}^{\alpha }-{\left(h_{ij}^{\alpha }\right)}^{2}\right]\\ & -\frac{1}{2}\sum_{\alpha =n+1}^{m}\;\sum_{1\leq i<j\leq n}\left[h_{ii}^{*\alpha }h_{jj}^{*\alpha }-{\left(h_{ij}^{*\alpha }\right)}^{2}\right]. \end{array}$$

By subtracting Equations (21) and (22) from Equation (23), we obtain

(24) $$\begin{array}{c} \left(\tau -K\left(\pi_{1}\right)-K\left(\pi_{2}\right)\right)-\left(\tau_{0}-K_{0}\left(\pi_{1}\right)-K_{0}\left(\pi_{2}\right)\right)\geq \left(n^{2}-n-4\right)\frac{c}{4}-\\ -\frac{1}{2}\sum_{\alpha =n+1}^{m}\left(\sum_{1\leq i<j\leq n}h_{ii}^{\alpha }h_{jj}^{\alpha }-h_{11}^{\alpha }h_{22}^{\alpha }-h_{33}^{\alpha }h_{44}^{\alpha }\right)\\ -\frac{1}{2}\sum_{\alpha =n+1}^{m}\left(\sum_{1\leq i<j\leq n}h_{ii}^{*\alpha }h_{jj}^{*\alpha }-h_{11}^{*\alpha }h_{22}^{*\alpha }-h_{33}^{*\alpha }h_{44}^{*\alpha }\right). \end{array}$$

Let *H* and $H^{*}$ denote the mean curvature vectors with respect to the dual connections ∇ and $\nabla^{*}$, respectively. Then, the above lemma implies

$$\begin{array}{c} \sum_{1\leq i<j\leq n}h_{ii}^{\alpha }h_{jj}^{\alpha }-h_{11}^{\alpha }h_{22}^{\alpha }-h_{33}^{\alpha }h_{44}^{\alpha }\leq \frac{n-3}{2(n-2)}{\left(\sum_{i=1}^{n}h_{ii}^{\alpha }\right)}^{2}=\frac{n^{2}\left(n-3\right)}{2(n-2)}{\left(H^{\alpha }\right)}^{2},\\ \sum_{1\leq i<j\leq n}h_{ii}^{*\alpha }h_{jj}^{*\alpha }-h_{11}^{*\alpha }h_{22}^{*\alpha }-h_{33}^{*\alpha }h_{44}^{*\alpha }\leq \frac{n-3}{2(n-2)}{\left(\sum_{i=1}^{n}h_{ii}^{*\alpha }\right)}^{2}=\frac{n^{2}\left(n-3\right)}{2(n-2)}{\left(H^{*\alpha }\right)}^{2}. \end{array}$$

By summing the two above relations and substituting the result into Equation (24), we get

$$\tau -K\left(\pi_{1}\right)-K\left(\pi_{2}\right)\geq \tau_{0}-K_{0}\left(\pi_{1}\right)-K\left(\pi_{2}\right)+\left(n^{2}-n-4\right)\frac{c}{4}-\frac{n^{2}\left(n-3\right)}{4(n-2)}{(∥H∥}^{2}+∥H^{*}{∥}^{2}).$$

In summery, we may state our main result.

**Theorem** **1.** *Let $M^{n}$(n≥4) be a statistical submanifold in a Hessian manifold ${\tilde{M}}^{m}\left(c\right)$ of constant Hessian curvature c. Then, for any $p\in M^{n}$ and any plane sections $\pi_{1}$ and $\pi_{2}$ at p, we have*

$$\tau_{0}-K_{0}\left(\pi_{1}\right)-K_{0}\left(\pi_{2}\right)\leq \tau -K\left(\pi_{1}\right)-K\left(\pi_{2}\right)+\frac{n^{2}\left(n-3\right)}{4(n-2)}{(∥H∥}^{2}+∥H^{*}{∥}^{2})-\left(n^{2}-n-4\right)\frac{c}{4},$$

*where $\tau_{0}$ and $K_{0}$ are the scalar curvature and the sectional curvature of $M^{n}$ with respect to the Riemann curvature tensor and τ and K with respect to the Hessian curvature tensor Q.*

*Moreover, the equality holds if and only if for any α∈{n+1,…,m},*

$$\begin{array}{c} h_{11}^{\alpha }+h_{22}^{\alpha }=h_{33}^{\alpha }+h_{44}^{\alpha }=h_{55}^{\alpha }=\ldots =h_{nn}^{\alpha },\\ h_{11}^{*\alpha }+h_{22}^{*\alpha }=h_{33}^{*\alpha }+h_{44}^{*\alpha }=h_{55}^{*\alpha }\ldots =h_{nn}^{*\alpha },\\ h_{ij}^{\alpha }=h_{ij}^{*\alpha }=0,\;\forall 1\leq i\neq j\leq n. \end{array}$$

An immediate consequence of Theorem 1 is the following.

**Theorem** **2.** *Let $M^{n}$(n≥4) be a statistical submanifold in a Hessian manifold ${\tilde{M}}^{m}\left(c\right)$ of constant Hessian curvature c. If there exist a point $p\in M^{n}$ and two mutually orthogonal plane sections $\pi_{1}$ and $\pi_{2}$ at p such that*

$$\left(\tau -K\left(\pi_{1}\right)-K\left(\pi_{2}\right)\right)-\left(\tau_{0}-K_{0}\left(\pi_{1}\right)-K_{0}\left(\pi_{2}\right)\right)<\left(n^{2}-n-4\right)\frac{c}{4},$$

*then $M^{n}$ is nonminimal in ${\tilde{M}}^{m}\left(c\right)$, i.e., either H≠0 or $H^{*}\neq 0$.*

Theorem 1 represents a δ(2,2) Chen inequality for statistical submanifolds in Hessian manifolds of constant Hessian curvature. 
